# Predicting pneumonia algorithm in stroke patients

**DOI:** 10.3389/fneur.2025.1690049

**Published:** 2025-11-19

**Authors:** Jong Weon Lee, Hyun-Joung Lee, Hyeon Ju Jang, Yeseul Yun, Deog Young Kim

**Affiliations:** 1 Department of Rehabilitation Medicine, Yonsei University College of Medicine, Seoul, Republic of Korea; 2 Research Institute of Rehabilitation Medicine, Yonsei University College of Medicine, Seoul, Republic of Korea; 3 Department of Speech-Language Pathology, Wonkwang Digital University, Seoul, Republic of Korea; 4 Research Institute for Future Medicine, Samsung Medical Center, Seoul, Republic of Korea

**Keywords:** stroke, pneumonia, aspiration, cough, deglutition disorders

## Abstract

**Background:**

Pneumonia is a serious complication of stroke, particularly in patients with dysphagia during inpatient rehabilitation, as it significantly increases morbidity, prolongs hospital stays, and impairs functional recovery. Early identification of patients at risk for pneumonia is crucial for improving outcomes and reducing post-stroke complications. This study aimed to develop a comprehensive algorithm for predicting post-stroke pneumonia risk by integrating clinical assessments of defense mechanisms against pneumonia.

**Methods:**

This case-control study enrolled stroke patients at a single tertiary hospital and followed them for 4 weeks to assess pneumonia incidence. A total of 812 patients aged 20 years or older with ischemic or hemorrhagic stroke and signs of dysphagia were screened. Of these, 484 were excluded based on the following criteria: inability to maintain a sitting posture with back support, dyspnea requiring oxygen supplementation, concurrent aspiration pneumonia before enrollment, infectious diseases requiring isolation, and refusal to participate. Final cohort of 328 patients was enrolled. All participants underwent evaluations, including a videofluoroscopic swallowing study (VFSS), a modified cough reflex test (mCRT), and assessments of nutritional status (serum albumin) and cognitive function [Mini-Mental State Examination (MMSE)]. Pneumonia was diagnosed using the Mann criteria, and predictive factors were analyzed using univariate logistic regression and classification and regression tree (CART) analysis.

**Results:**

Among 328 participants, 28 (8.5%) developed pneumonia. Significant predictors included tracheostomy status (OR 9.34), VFSS-confirmed aspiration (OR 8.21) and bilateral stroke lesions (OR 5.91). CART analysis revealed tracheostomy, VFSS-confirmed aspiration, cough frequency, albumin levels, and MMSE scores as key predictors. The algorithm demonstrated a predictive accuracy of 92.7% with an AUC of 0.89 (95% CI: 0.82–0.95).

**Conclusion:**

This study developed a highly accurate predictive algorithm for post-stroke pneumonia, emphasizing the role of defense mechanisms against pneumonia. Implementing this algorithm in clinical practice could enable early preventive measures, reduce pneumonia incidence, and improve patient outcomes.

## 1 Introduction

Pneumonia is a serious complication following stroke, with an estimated incidence of 5%–30% (1, 2). Subsequent treatments for pneumonia significantly prolong hospital stays, increase medical costs, hinder functional recovery, and lead to poor outcomes (3–5). Therefore, early identification of patients at risk is essential not only for preventing post-stroke pneumonia but also for optimizing recovery.

Several risk scoring tools such as the A^2^DS^2^ score (6), acute ischemic stroke-associated pneumonia score (AIS-APS) (7), and independence pre-stroke, sex, age, National Institutes of Health Stroke Scale (ISAN) score (8) use clinical factors such as age, sex, stroke severity, and comorbidities to predict pneumonia risk. However, these tools primarily focus on pneumonia in the acute stage of stroke and do not directly assess the body’s defense mechanisms against infection. Instead, they rely on simplified combinations of demographic factors, stroke severity, and medical history, despite the fact that pneumonia develops when pathogens reach the alveoli and overwhelm host defenses due to the microorganism’s virulence or inoculum` size.

Understanding the complex defense mechanisms against pneumonia is crucial for accurately predicting post-stroke pneumonia risk. Emerging evidence suggests that multiple neuroanatomical (9) and physiological defense mechanisms–such as airway clearance (10), swallowing function (11), cognitive processing (12), and immunonutrition (13)–play critical roles in pneumonia prevention. These defense mechanisms function in an integrated manner rather than independently, yet their interconnections remain poorly understood. Therefore, we hypothesize that integrating standardized assessment tools that evaluate each level of the neurophysiologic defense system against pneumonia will improve the prediction of post-stroke pneumonia compared to existing models.

## 2 Materials and methods

### 2.1 Study participants and data collection

**Figure 1:**
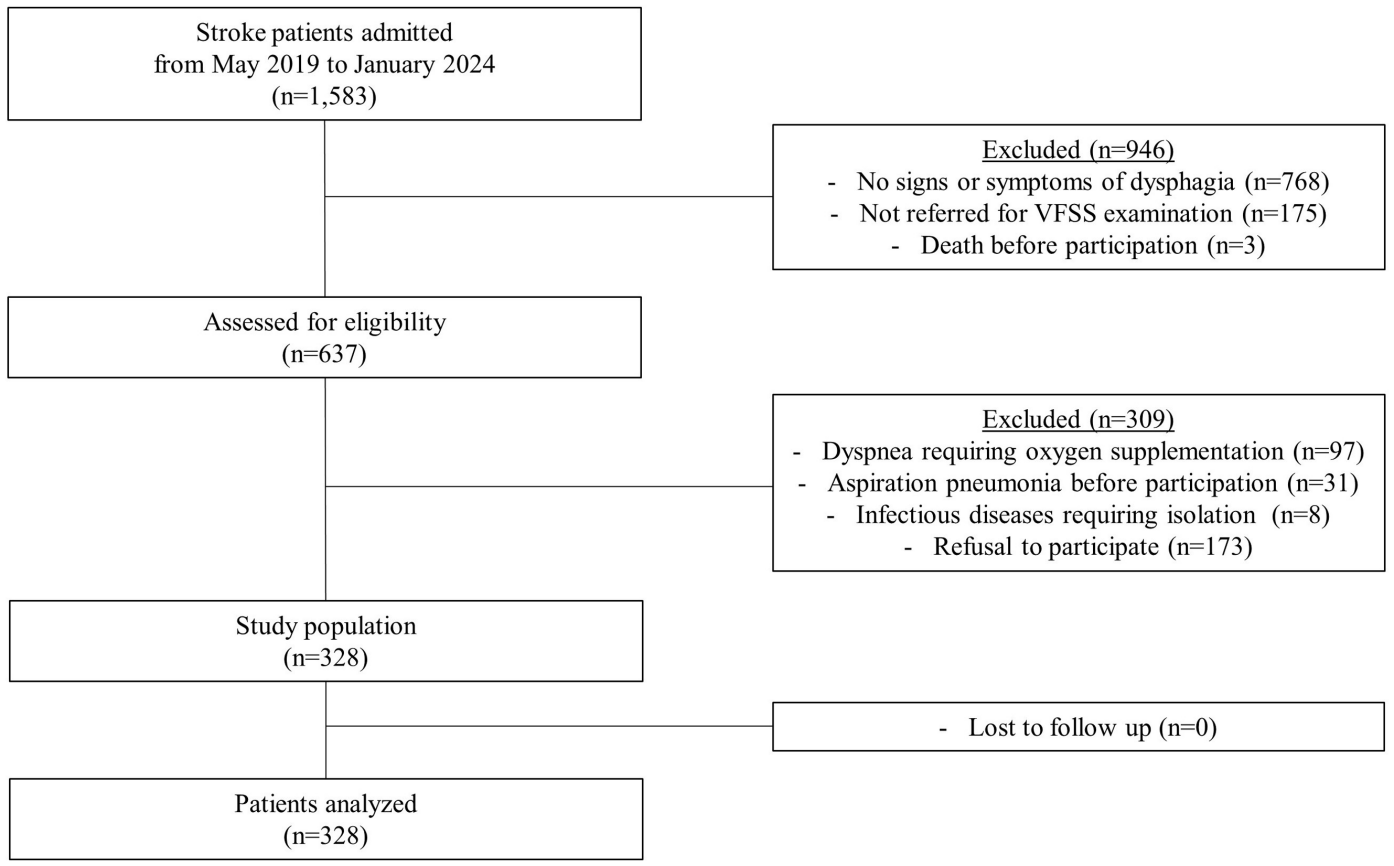
Flowchart of the study participants.

In this prospective case-control study, stroke patients who were admitted to a single tertiary hospital in Korea from 2019 to 2024 were screened based on the following inclusion criteria: (1) either ischemic or hemorrhagic stroke confirmed by computed tomography or magnetic resonance imaging, (2) age 20 years or older, and (3) presence of signs or symptoms of dysphagia, and (4) referred for videofluoroscopic swallowing study (VFSS). The exclusion criteria were as follows: (1) dyspnea requiring oxygen supplementation, (2) a prior diagnosis of aspiration pneumonia before enrollment, (3) a diagnosis of an infectious disease requiring isolation, and (4) refusal to participate. A total of 328 patients were enrolled, with no loss to follow-up (Figure 1).

The required sample size was determined using Power Analysis and Sample Size Software [PASS (version 12, NCSS, Kaysville, Utah, USA)], based on an estimation of two proportions. To assess the utility of the evaluation tools through regression analysis, with a significance level of 0.05, a power of 0.8, and accounting for a 10% dropout rate, the target sample size was calculated as 328.

All 328 patients underwent VFSS and modified cough reflex test (mCRT). Demographic information and baseline characteristics were collected at admission. Neurological and laboratory assessments, including the Mini-Mental State Examination (MMSE), serum albumin levels, white blood cell (WBC) count, total neutrophil count, neutrophil-to-lymphocyte ratio (NLR), and C-reactive protein (CRP) level, were conducted within 24 h of admission. Written informed consent was obtained from all participants or their legally authorized representatives and the study was performed on accordance with the Declaration of Helsinki. The study protocol was approved by the Institutional Review Board of our hospital (IRB No. 4-2019-0111).

### 2.2 Clinical assessment of dysphagia

Patients were assessed for dysphagia by certified rehabilitation specialists and trained speech-language pathologists. As documented in previous studies (14–16), dysphagia was defined by the presence of any of the following: impaired sensory or motor function of oral structures (jaw, lips, tongue, palate, or cheeks), cranial nerve dysfunction (trigeminal, facial, glossopharyngeal, vagus, or hypoglossal nerves), and voice changes (wet or gurgly voice) after swallowing, drooling, or apraxia.

### 2.3 Definition of pneumonia

Patients were monitored for 4 weeks following baseline assessments, and pneumonia was diagnosed based on the Mann criteria (17). A blinded physician confirmed the diagnosis if three or more of the following criteria were met: fever (>38 °C), productive cough with purulent sputum, abnormal respiratory findings (tachypnea >22/min, tachycardia, inspiratory crackles, or bronchial breathing), abnormal chest radiographic findings, arterial hypoxemia (PO₂ < 70 mmHg or SpO₂ < 94%), or identification of a relevant pathogen (positive Gram stain or culture).

Based on previous studies, pneumonia risk was classified into three categories. High risk was defined as a pneumonia incidence exceeding 25% (8, 18). No risk was assigned to incidences below 2%, a threshold lower than the lowest reported post-stroke pneumonia incidence (2.3%) in previous studies (18, 19). Incidences between 2% and 25% were categorized as low risk.

### 2.4 Videofluoroscopic swallowing study

VFSS, the gold standard for evaluating swallowing function and visualizing oral-pharyngeal anatomy, was used to detect aspiration (5). A real-time fluoroscopic video was recorded with patients seated upright in a chin-tuck position. A barium-impregnated bolus was prepared in both plain liquid and semisolid yogurt mixed with a liquid thickener. Boluses were administered in a fixed order, starting with semisolid followed by liquid, with each consistency tested at volumes of 5 and 15 mL. The test was discontinued if aspiration was observed or if the patient was unable to tolerate the procedure. All 328 participants completed the VFSS protocol.

Penetration or aspiration was assessed using the 8-point Penetration-Aspiration Scale (PAS) (20), which evaluates the depth of airway invasion and whether the material is expelled. A PAS score greater than 5 was considered indicative of aspiration, as supported by previous studies (21). VFSS recordings were reviewed and PAS scores were provided by rehabilitation specialists blinded to the patients’ clinical data.

### 2.5 Modified cough reflex test

A Sidestream nebulizer (Philips Respironics, Parsippany, NJ, USA) was modified to include a peak flow meter via a T-piece. Capsaicin (Sigma-Aldrich Korea, Seoul, Korea) was dissolved in polyoxyethylene sorbitan and ethanol, then diluted with normal saline to concentrations of 7.8 μM and 31.25 μM. Patients inhaled 7.8 μM capsaicin mist through the nebulizer for 15 s. For those with a tracheostomy tube, the mist was administered directly through the tracheostomy site. The total number of coughs and the peak cough flow (PCF) were recorded within 30 s after inhalation. The procedure was then repeated using the 31.25 μM capsaicin solution. Four of 328 participants (1.2%) were unable to complete the mCRT due to poor cooperation.

### 2.6 Statistical analysis

Numerical variables are presented as mean with standard deviation and categorical variables as count with percentage. The Mann–Whitney test was used to compare continuous variables, and the chi-square test was applied to compare categorical variables between the pneumonia and non-pneumonia groups. Univariate logistic regression was conducted to identify potential risk factors for pneumonia. Variables with *p* < 0.01 were included in the classification and regression tree (CART) analysis. CART analysis was performed using the “gini” index, with a maximum tree depth of 3 and complexity parameter of 0.001. Minimum split size and bucket size were set at 4 and 2, respectively. Ten-fold cross-validation was repeated twice to evaluate performance, and the area under the receiver operating characteristic curve (AUC) and overall accuracy were calculated. For analyses involving mCRT, four missing data were excluded. A *p*-value of less than 0.05 was considered statistically significant. All analyses were conducted using R statistical software (version 4.2.3, R Project for Statistical Computing, Vienna, Austria) and SPSS (version 25.0, IBM, Chicago, Illinois, USA).

## 3 Results

### 3.1 Patient characteristics

Among the 328 participants, 28 (8.5%) were diagnosed with pneumonia within 4 weeks (Table 1). The age of the participants was 63.4(14.4) years, and 40.5% were female. The pneumonia group had a significantly higher proportion of patients with tracheostomy (53.6% vs. 11.0%), aspiration on VFSS (71.4% vs. 23.5%), and stroke lesions affecting both brain hemispheres (64.3% vs. 23.3%) than the non-pneumonia group (*p* < 0.01). In mCRT, the pneumonia group exhibited significantly lower cough frequency at both 7.8 μM [1.6 (2.5) vs. 4.3 (4.6)] and 31.25 μM [2.7 (3.6) vs. 6.3 (5.7)], as well as lower PCF at 31.25 μM [45.0 (74.4) L/min vs. 82.0 (131.3) L/min] (*p* < 0.01). The pneumonia group also had significantly lower albumin levels [3.6 (0.4) g/dL vs. 4.0 (0.4) g/dL], MMSE scores [8.2 (9.4) vs. 18.4 (10.3)], and total lymphocyte counts [1.5 (0.4) × 10^3^/μL vs. 1.7 (0.6) × 10^3^/μL] (*p* < 0.01). However, no significant differences were observed between the two groups in stroke types, lesion locations, or biomarkers such as WBC, neutrophil counts, NLR, and CRP.

**Table 1:** Baseline characteristics of the study population.

Characteristics	Total (*n* = 328)	Pneumonia (+) (*n* = 28)	Pneumonia (−) (*n* = 300)	*p*-value
Age, mean (SD)	63.4 (14.4)	67.5 (12.1)	63.0 (14.6)	0.120
Women, no. (%)	133 (40.5)	8 (28.6)	125 (41.7)	0.251
MMSE, mean (SD)	17.5 (10.6)	8.2 (9.4)	18.4 (10.3)	<0.001
Tracheostomy status, no. (%)	48 (14.6)	15 (53.6)	33 (11.0)	<0.001
Stroke duration until VFSS, mean (SD), days	111.2 (218.4)	76.1 (52.9)	114.5 (227.6)	0.021
Death, no. (%)	1 (0.3%)	1 (3.6%)	0 (0.0%)	0.137
Involved hemisphere, no. (%)				<0.001
Hemi side	240 (73.2)	10 (35.7)	230 (76.7)	
Both sides	88 (26.8)	18 (64.3)	70 (23.3)	
Stroke type, no. (%)				0.627
Ischemic	183 (55.8)	16 (57.1)	167 (55.7)	
Hemorrhagic	140 (42.7)	11 (39.3)	129 (43.0)	
Both	5 (1.5)	1 (3.6)	4 (1.3)	
Tentorial region, no. (%)				0.240
Supratentorial	261 (79.6)	20 (71.4)	241 (80.3)	
Infratentorial	52 (15.9)	5 (17.9)	47 (15.7)	
Both	15 (4.6)	3 (10.7)	12 (4.0)	
VFSS PAS ≥ 6, no. (%)	90 (27.6)	20 (71.4)	70 (23.5)	<0.001
Modified cough reflex test				
Frequency @7.8 μM, mean (SD)	4.1 (4.5)	1.6 (2.5)	4.3 (4.6)	<0.001
Frequency @31.25 μM, mean (SD)	6.0 (5.6)	2.7 (3.6)	6.3 (5.7)	<0.001
PCF @7.8 μM, mean (SD), L/min	56.9 (105.0)	43.1 (105.8)	58.2 (105.0)	0.467
PCF @31.25 μM, mean (SD), L/min	78.9 (127.8)	45.0 (74.4)	82.0 (131.3)	0.028
WBC, mean (SD), ×10^3^/μL	7.2 (2.1)	7.4 (1.5)	7.2 (2.2)	0.406
Neutrophil, mean (SD), ×10^3^/μL	4.7 (1.9)	5.0 (1.3)	4.7 (1.9)	0.175
Lymphocyte, mean (SD), ×10^3^/μL	1.7 (0.6)	1.5 (0.4)	1.7 (0.6)	0.004
NLR, mean (SD)	3.2 (2.1)	3.8 (1.9)	3.2 (2.1)	0.160
CRP, mean (SD), mg/L	8.1 (15.1)	13.1 (11.4)	7.6 (15.3)	0.062
Albumin, mean (SD), g/dL	4.0 (0.4)	3.6 (0.4)	4.0 (0.4)	<0.001

MMSE, Mini-Mental State Examination; VFSS, videofluoroscopic swallowing study; PAS, Penetration-Aspiration Scale; PCF, peak cough flow; WBC, white blood cell count; NLR, neutrophil-to-lymphocyte ratio; CRP, C-reactive protein.

### 3.2 Clinical predictors of post-stroke pneumonia

Univariate logistic regression analysis identified several significant predictors of post-stroke pneumonia (Table 2, *p* < 0.01). Tracheostomy status had the highest odds ratio (OR) at 9.34 (95% CI: 4.10–21.65), followed by aspiration on VFSS (OR: 8.21) and bilateral stroke involvement (OR: 5.91). MMSE scores, cough frequency at both 7.8 μM and 31.25 μM mCRT, and albumin levels also showed statistically significant associations. However, age and sex were not significantly associated with post-stroke pneumonia, and PCF at both 7.8 μM and 31.25 μM mCRT did not demonstrate significant ORs.

**Table 2:** Clinical predictors of post-stroke pneumonia: univariate logistic regression analysis.

Variables	Univariable analysis	
	OR (95% CI)	*p*-value
Age	1.024 (0.994–1.055)	0.122
Sex		
Men	Reference	
Women	0.560 (0.239–1.312)	0.182
MMSE	0.913 (0.875–0.949)	<0.001
Tracheostomy		
(−)	Reference	
(+)	9.336 (4.096–21.648)	<0.001
Stroke duration until VFSS	0.998 (0.993–1.001)	0.324
Involved hemisphere		
Hemi side	Reference	
Both	5.914 (2.658–13.881)	<0.001
Stroke type		
Ischemic	Reference	
Hemorrhagic	0.890 (0.399–1.983)	0.776
Both	2.609 (0.275–24.770)	0.404
Tentorial region		
Supratentorial	Reference	
Infratentorial	1.282 (0.458–3.586)	0.636
Both	3.012 (0.785–11.561)	0.108
VFSS PAS		
<6	Reference	
≥6	8.214 (3.587–20.587)	<0.001
Modified cough reflex test		
Frequency @7.8 μM	0.795 (0.681–0.929)	0.004
Frequency @31.25 μM	0.520 (0.766–0.947)	0.003
PCF @7.8 μM	0.998 (0.993–1.003)	0.468
PCF @31.25 μM	0.995 (0.989–1.001)	0.135
WBC	1.056 (0.879–1.241)	0.534
Neutrophil	1.101 (0.901–1.313)	0.307
Lymphocyte	0.999 (0.998–1.000)	0.050
NLR	1.109 (0.943–1.275)	0.166
CRP	1.015 (0.995–1.035)	0.101
Albumin	0.069 (0.024–0.198)	<0.001

MMSE, Mini-Mental State Examination; VFSS, videofluoroscopic swallowing study; PAS, Penetration-Aspiration Scale; PCF, peak cough flow; WBC, white blood cell count; NLR, neutrophil-to-lymphocyte ratio; CRP, C-reactive protein.

### 3.3 Construction of decision tree with classification and regression tree analysis

**Figure 2:**
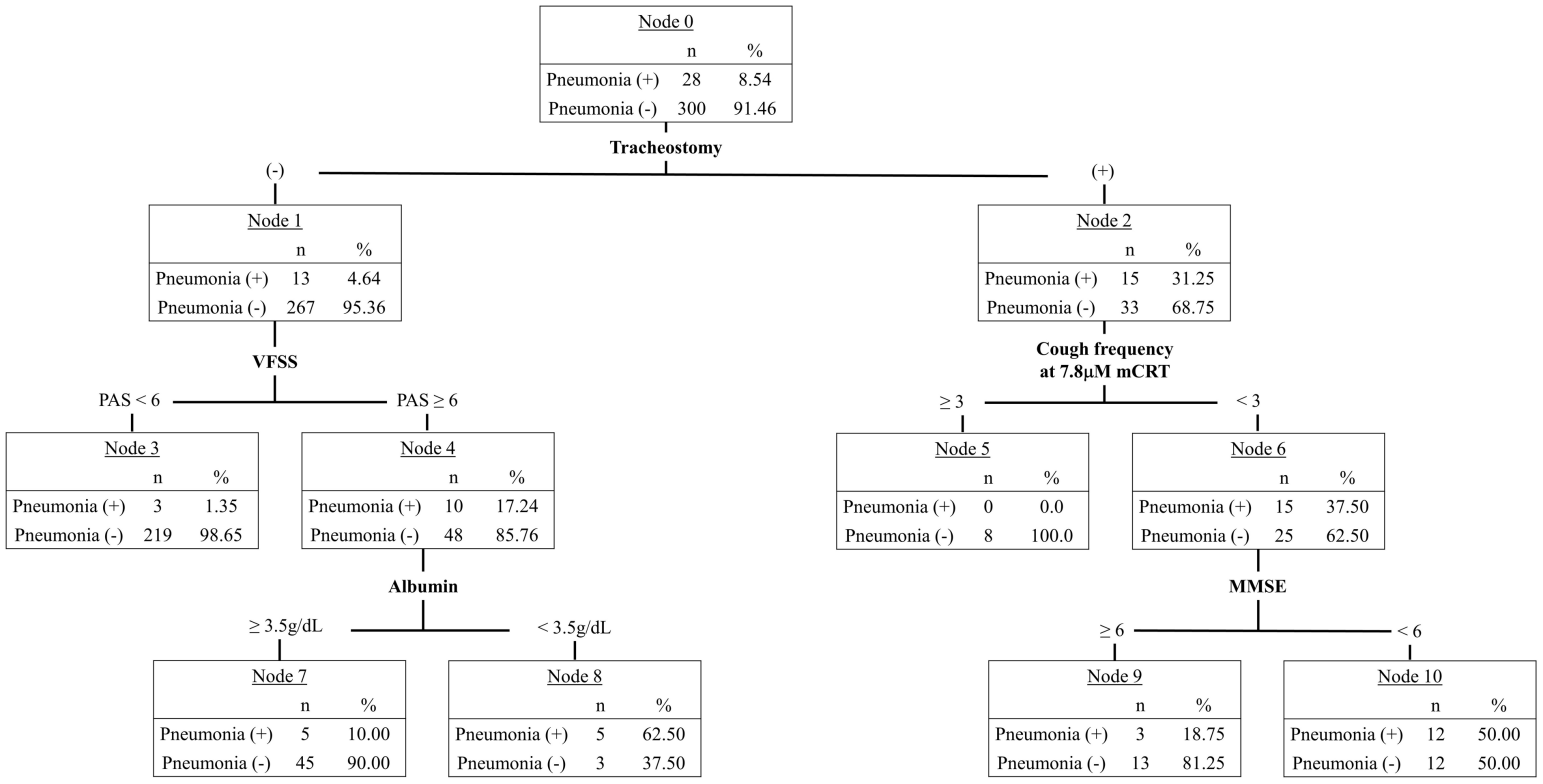
Classification and regression tree (CART) analysis identifying factors predicting post-stroke pneumonia.

Significant clinical factors associated with post-stroke pneumonia in the logistic regression analysis were included in the CART analysis. Tracheostomy status, aspiration on VFSS, cough frequency at 7.8 μM mCRT, albumin levels, and MMSE scores were identified as significant predictors of post-stroke pneumonia (Figure 2). However, cough frequency at 31.25 μM mCRT and bilateral hemispheric lesions were not utilized in constructing the decision tree.

Tracheostomy status was the primary predictor in the decision tree, with 31.25% (*n* = 15) of patients with tracheostomy developing pneumonia, compared with 4.64% (*n* = 13) of those without. Among patients without tracheostomy, a PAS score below 6 on VFSS indicated a pneumonia incidence of 1.35% (*n* = 3). If aspiration was confirmed on VFSS, low nutritional status—defined by an albumin level below 3.5 g/dL—was associated with a 62.50% (*n* = 5) pneumonia risk, whereas an albumin level of 3.5 g/dL or higher indicated a 10.0% (*n* = 5) risk. In patients with tracheostomy, a cough frequency of 3 or higher at 7.8 μM mCRT was associated with no pneumonia risk. However, if cough frequency was below 3, an MMSE score of less than 6 resulted in a 50.0% (*n* = 12) pneumonia incidence, while a score of 6 or higher indicated an 18.75% (*n* = 3) risk.

**Figure 3:**
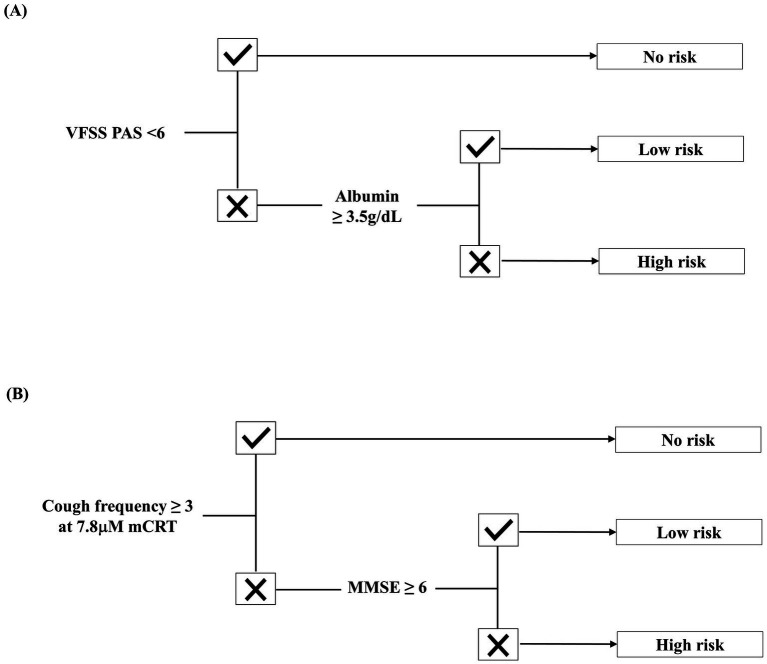
Predictive algorithm for aspiration pneumonia in post-stroke patients with dysphagia. **(A)** Algorithm for patients without tracheostomy. **(B)** Algorithm for patients with tracheostomy.

The predictive accuracy of the algorithm (Figure 3) was 92.7%, with an AUC of 0.89 (95% CI: 0.82–0.95). In the no-risk group, the algorithm underestimated pneumonia risk for three patients who were later diagnosed with pneumonia. However, overall accuracy for the no-risk group remained high at 98.7% (227/230).

## 4 Discussion

This study is the first to identify predictors and develop an algorithm for forecasting pneumonia risk in stroke patients undergoing inpatient rehabilitation using defense mechanism-specific assessment tools. The key predictors incorporated into the algorithm include tracheostomy status, aspiration on VFSS, cough frequency, albumin levels, and MMSE scores. This integrated algorithm demonstrated a predictive accuracy of 92.7%, with an AUC of 0.89. Notably, this AUC tended to be higher than that of previously developed predictive models, such as the A^2^DS^2^ (AUC 0.84), AIS-APIS (AUC 0.78), and ISAN scores (AUC 0.79) (6–8), which primarily rely on general clinical characteristics.

Understanding the multifaceted defense mechanisms against pneumonia is critical to advancing predictive models. First, anatomic protection against aspiration is ensured through anatomic integrity, glottic closure and activation of oropharyngeal muscles (11). Second, the voluntary cough reflex clears foreign material from the airway and serves as a primary line of defense (10). Third, nutritional status plays a role in immune competence, with malnutrition associated with systemic inflammation and impaired host defense (13). Fourth, the act of swallowing involves complex cortical and subcortical processing, underscoring the cognitive contribution to airway protection (12). This study’s findings support the notion that a multi-dimensional evaluation of these defense layers can significantly enhance predictive capability.

In this study, tracheostomy status emerged as the most influential predictor in the decision tree and the factor with the highest OR. Aspiration rates in tracheostomized patients have been reported to range from 50% to 83% (22, 23). This finding aligns with previous study results showing tracheostomy as a major contributor to increased post-stroke pneumonia risk (24, 25). Dai et al. (24) demonstrated that prolonged tracheostomy duration is associated with a higher risk of pneumonia in stroke patients with both tracheostomy and dysphagia. Despite its role in secretion management, tracheostomy negatively affects airway protection mechanisms. The tracheostomy tube anchors the larynx to surrounding neck tissues, restricting laryngeal elevation (25), reducing protective laryngeal reflexes, and causing uncoordinated laryngeal closure responses (26). Moreover, it shortens the duration of vocal cord adduction/abduction (27), leading to disuse atrophy of laryngeal muscles (28). Additionally, an open tracheostomy reduces subglottic air pressure during swallowing, further compromising airway protection (29). These previous findings, along with the present study, reinforce tracheostomy as a critical risk factor for post-stroke pneumonia.

In this study, aspiration detected in VFSS was the second strongest predictor of pneumonia, aligning with findings from Martino et al. (30), who reported an 11-fold increased risk of pneumonia in stroke patients with confirmed aspiration. However, in the proposed algorithm, VFSS-confirmed aspiration was not used for patients with tracheostomy. This is consistent with the findings of Dai et al. (24), who found that in post-stroke patients with tracheostomy, only total tracheostomy duration, cough reflex dysfunction, and oropharyngeal phase dysfunction were significant pneumonia risk factors in logistic regression analysis, despite significant differences in PAS scores in descriptive statistics. In this study, aspiration in VFSS was observed in 66.7% (32/48) of tracheostomized patients, suggesting that its high incidence may limit the predictive value of VFSS-confirmed aspiration in this population.

Cough frequency also played a significant role in predicting pneumonia risk. A cough frequency of three or more was associated with a lower risk of pneumonia in tracheostomized patients. Previous studies have reported that cough frequency in the cough reflex test has a sensitivity of 0.70 to 0.87 for detecting silent aspiration in stroke patients (31, 32). Pekacka-Egli et al. (33) speculated that low cough frequency may indicate diminished cough sensitivity and reduced respiratory tract protection, increasing pneumonia risk due to silent aspiration. Further, Trimble et al. (34) and Miles et al. (35) proposed that fewer than two coughs in the cough reflex test could serve as a criterion for screening silent aspiration. Perry et al. (36) recommended assessing cough frequency as an initial screening measure before proceeding with oral intake trials to mitigate aspiration pneumonia risk in stroke patients with dysphagia. Consistent with prior study results, findings from this study underscore the importance of cough frequency in predicting pneumonia risk, particularly in tracheostomized patients who are highly susceptible to silent aspiration.

However, PCF did not show a significant association with pneumonia risk in this study. Kulnik et al. (37) reported that a strong cough protects against aspiration-related pneumonia and that stroke patients with a PCF below 400 L/min have a three-fold increased risk of pneumonia. Min et al. (38) and Bianchi et al. (10) suggested PCF as a useful prognostic factor for aspiration risk in patients with dysphagia. However, in this study, PCF at both 7.8 μM and 31.25 μM mCRT did not significantly predict post-stroke pneumonia. This discrepancy may be due to challenges in accurately measuring PCF in patients with tracheostomy and cognitive impairments, which may have led to generally low PCF values.

In this study, severe cognitive impairment was associated with a higher risk of pneumonia in tracheostomized patients. While earlier studies focused on the laterality (16) or locations (39) of brain lesions affecting dysphagia, recent research has emphasized the degree (40) and neuropsychological profiles (12) of cognitive impairment. Davenport et al. (41) suggested that sensory recognition of airway irritation requires cognitive awareness, enabling compensatory responses when the cough reflex is impaired. The algorithm in Figure 3B aligns with this rationale, incorporating cognitive function as a secondary defense mechanism when the cough reflex is compromised.

Malnutrition was another key factor associated with increased pneumonia risk, consistent with previous findings (13, 42–44). Dysphagia in stroke patients is closely associated with malnutrition due to reduced nutrient intake and increased metabolic demands during the recovery phase of stroke (42, 45). Furthermore, malnutrition weakens immune function and is strongly associated with infection, particularly post-stroke pneumonia, independent of aspiration (43, 44). The FOOD trial demonstrated that pneumonia was more common in undernourished stroke patients due to the detrimental effects of poor nutrition on immune function (13). More recently, a meta-analysis by Chen et al. found that proper nutrition in stroke patients may reduce systemic inflammation, enhance immune response, and subsequently lower the risk of stroke-associated infections (46). Figure 3A supports these findings, emphasizing the role of nutrition in pneumonia prevention, even in the presence of aspiration.

This integrative algorithm underscores the importance of evaluating multiple protective mechanisms against pneumonia—anatomical protection, swallowing reflex, cough reflex, cognition, and immunonutrition—to enhance predictive accuracy. A key distinction between this algorithm and previous predictive models is the lack of significance of age and sex in predicting post-stroke pneumonia. While earlier models (6–8) incorporated these demographic factors, a recent meta-analysis by Guo et al. (47) suggested that gender does not influence pneumonia risk, and many risk scoring tools (7, 48, 49) have excluded sex from their scoring systems. Although age is consistently identified as a risk factor, the arbitrary cutoff points used across different scoring systems limit its reliability. Consistent with recent studies, neither age nor sex demonstrated significant predictive value in this study.

This study has several strengths. First, it employs highly specific assessment tools to evaluate defense mechanisms against pneumonia. Second, the predictive accuracy of this algorithm (AUC 0.89) tended to be higher than that of previous demographic- and clinically based models (AUC 0.79–0.84) (6–8). Third, most clinical factors included in the algorithm are routinely obtainable and easy to assess, making it practical and accessible for widespread implementation.

Several limitations should be acknowledged. First, despite VFSS is the gold standard for detecting aspiration, not all patients can undergo this test because of patient’s condition or the limited availability of VFSS at many institutions. Incorporating simplified bedside screening tests, such as the water swallow test, could improve accessibility in future studies. Second, the incidence of pneumonia (*n* = 28) and the number of tracheostomized patients (*n* = 48) was relatively small. Moreover, higher-risk populations, such as patients requiring supplemental oxygen or with active pneumonia, were excluded. Future multicenter studies with larger sample size that include these higher-risk cohorts will be needed to validate it. Third, while dysphagia was clinically assessed by certified rehabilitation specialists and trained speech-language pathologists, standardized dysphagia scales–such as the Gugging Swallowing Screen (GUSS) (50) and the Mann Assessment of Swallowing Ability (MASA) (51)–were not applied. Adopting such validated tools would enable more consistent and reproducible assessment of swallowing, facilitate comparability across studies, and strengthen the clinical applicability of the predictive model. Fourth, this model was not compared head-to-head in the same cohort and use different case mixes and predictor sets with previously developed predicting models. Although higher AUC in this model is encouraging, prospective head-to-head comparison and external validation with same cohort is warranted. Finally, oral hygiene may be a potential predictor of post-stroke pneumonia, as bacterial colonization in saliva contributes to aspiration pneumonia. However, this data was not collected due to the lack of standardized assessment tools.

## 5 Conclusion

This study was the first prospective study to develop an algorithm for predicting post-stroke pneumonia incidence using defense mechanism-specific assessment tools during inpatient rehabilitation. Among the various factors associated with aspiration pneumonia, tracheostomy status, aspiration in VFSS, cough frequency, albumin levels, and cognitive function were identified as the key predictors. This algorithm offers a comprehensive framework for post-stroke pneumonia screening and may facilitate early preventive interventions for at-risk patients. Future studies with larger, more diverse samples-including higher-risk cohorts-and external multicenter validation will be needed before wider clinical implementation.

## Data Availability

The raw data supporting the conclusions of this article will be made available by corresponding author.
